# Spermatozoal sensitive biomarkers to defective protaminosis and fragmented DNA

**DOI:** 10.1186/1477-7827-5-36

**Published:** 2007-08-30

**Authors:** Roxani Angelopoulou, Konstantina Plastira, Pavlos Msaouel

**Affiliations:** 1 Experimental Embryology Unit, Department of Histology and Embryology, Medical School, National and Kapodistrian University of Athens, Greece

## Abstract

Human sperm DNA damage may have adverse effects on reproductive outcome. Infertile men possess substantially more spermatozoa with damaged DNA compared to fertile donors. Although the extent of this abnormality is closely related to sperm function, the underlying etiology of ensuing male infertility is still largely controversial. Both intra-testicular and post-testicular events have been postulated and different mechanisms have been proposed to explain the presence of damaged DNA in human spermatozoa. Three among them, i.e. abnormal chromatin packaging, oxidative stress and apoptosis, are the most studied and discussed in the present review. Furthermore, results from numerous investigations are presented, including our own findings on these pathological conditions, as well as the techniques applied for their evaluation. The crucial points of each methodology on the successful detection of DNA damage and their validity on the appraisal of infertile patients are also discussed. Along with the conventional parameters examined in the standard semen analysis, evaluation of damaged sperm DNA seems to complement the investigation of factors affecting male fertility and may prove an efficient diagnostic tool in the prediction of pregnancy outcome.

## Background

Sperm chromatin maturity and DNA integrity are necessary prerequisites for the completion of fertilization and subsequent embryo development [1]. Although an important level of sperm nuclear DNA damage can be detected in men with normal semen parameters, this is higher in infertile patients attending reproductive clinics and prominent among barely fertile older men [2]. Sperm DNA damage may be attributed to abnormal chromatin packaging, oxidative stress or poor DNA integrity [3]. Many studies combining standard semen analysis data with direct or indirect methods for the assessment of sperm DNA integrity and the evaluation of the DNA Fragmentation Index (DFI) from sperm used in ART, showed that there are threshold values of sperm DNA damage, beyond which embryo development and pregnancy outcome are severely compromised [4-12]. Furthermore, sperm DNA damage thresholds have been proposed in assays applied to *in vivo *conditions [4,5,13-15]. Thus, the clinician is guided as to the therapeutic approach that should be followed for the treatment of male infertility, according to the level of sperm chromatin maturity and DNA integrity found in the ejaculate [16].

Following a standard semen analysis, men are usually classified as infertile, sub-fertile or fertile and the World Health Organization (WHO) has suggested a range of arbitrary threshold values for human semen parameters, such as concentration, motility and morphology, to characterize infertility [17]. Because fertility is not only based on the absolute number of spermatozoa but also on their functional capability, additional methods exploring sperm DNA stability and integrity have been applied during the last decade to evaluate fertility disorders and to increase the predictive value of sperm analysis for procreation *in vivo *and *in vitro *[18]. With these new techniques it was shown that normozoospermic infertile men, in addition to those having poor semen parameters, have higher percentages of spermatozoa with DNA fragmentation compared to the individuals presenting with normal semen quality [19-23].

Sperm DNA fragmentation may result from aberrant chromatin packaging during spermiogenesis [23,24], defective apoptosis before ejaculation [25,26] or excessive production of reactive oxygen species (ROS) in the ejaculate [20,27,28]. Indeed, in a substantial percentage of infertile men (about 5%-15%) with complete protamine deficiency, the cause can be attributed to defective chromatin packaging [29]. Abortive apoptosis during spermatogenesis or deficient ligation of transient breaks during spermiogenesis may be another reason of sperm DNA damage [30]. Increased oxidative DNA damage and high levels of 8-hydroxy-2'-deoxyguanosine, a biomarker of oxidized DNA, have also been detected in the sperm of infertile men [19] and it has been reported that the likelihood of pregnancy has been found inversely correlated with 8-oxo-dG levels [31]. Furthermore, a positive correlation between sperm DNA fragmentation and ROS generation have been reported [32]. In a recent paper [3], the authors suggested that DNA damage of human spermatozoa is most commonly associated with high levels of ROS, mainly as a consequence of retained cytoplasmic droplets on the spermatozoon midpiece. This causes lipid peroxidation of cell membranes, due to the exposure to unsaturated fatty acids and high levels of oxidases, following spermiogenesis [33]. In addition, human spermatozoa DNA fragmentation may ensue, following an endonuclease cleavage [34], an activity also affected by oxidative stress. Therefore, the same cell could be damaged by both oxidative DNA stress and endonuclease-mediated DNA fragmentation [3].

Taking into account all these data, the present review examines abnormal chromatin packaging, oxidative stress and poor sperm DNA integrity as the possible causes of male infertility. It attempts to elaborate on results from numerous studies on these pathological conditions during spermatogenesis, spermiogenesis and in the ejaculated spermatozoa. Male genital tract infections and varicocele as aetiological factors in the generation of ROS and the consequences in sperm function and fertility are also discussed. Another chapter of this review elaborates on the crucial points of each specific technique and their validity on successful detection of DNA damage as well on the correlation of their data with fertilization, embryo development and maintenance of pregnancy. Preliminary data from our experiments on sperm from oligoasthnoteratozoospermic patients, examined with two of these techniques and discussion on the influence of age on these parameters are the conclusive points.

## Mechanisms

### Normal and defective protaminosis

During spermiogenesis (differentiation of the haploid spermatid to spermatozoon), the chromatin nucleosomal structure, seen in the interphase nucleus of round spermatids, is extensively modified. Most of the core somatic histones are replaced by sperm specific nuclear proteins, including transition proteins (TNPs), which are, in turn, replaced by the more basic protamines [29,35,36]. The transition proteins are synthesized before the deposition of the protamines. Four TNPs, TP1 to TP4, have been identified in humans [37]. These proteins by binding to DNA destabilize the nucleosomes and maintain the normal processing of protamines at the same time, thus promoting chromatin condensation [38-40].

Progression of sperm chromatin condensation evolves in a stepwise manner. The first biochemical change is the replacement of most of the somatic histones by testis variants [41]. Almost all known histone variants, including testis-specific histone members, are synthesized before and during the assembly of transition proteins and protamines and some are essential in the process of chromatin condensation and genome reorganization [42]. The initial phase of chromatin starts with hyperacetylation in the N-terminal histone tails and then other modifications cause dissociation of nucleosomes, while topoisomerase II unwinds DNA superhelicity. It is postulated that during chromatin packaging this endogenous nuclease (topoisomerase II) activity is important for the cleavage and then the ligation of nicks that facilitate protaminosis [43]. These nicks are thought to relieve torsional stress and aid chromatin rearrangement during the histone to protamine substitution [43]. Marcon & Boissonneault have clarified the mechanisms behind DNA folding and have demonstrated that this ephemeral DNA breakage occurs in parallel with chromatin remodeling, not only in mouse but also in human spermatids [44].

The mammalian TNPs are expressed at a high level at mid-spermiogenesis steps coinciding with chromatin remodeling and are involved in the repair of DNA single-strand breaks (SSB). TP1 can stimulate the repair of SSB *in vitro*, as well as the repair of UV-induced DNA lesions *in vivo*. The TP1 proteins can participate in the repair process following genotoxic insults and therefore they play an active role in maintaining the integrity of the male haploid [45]. After massive binding of TNPs to DNA, transcription ceases. Cessation of transcription is followed by the repair of DNA breaks and the beginning of the binding of phosphorylated protamines. These proteins are of low molecular mass and are highly basic [46]. Their phosphorylation facilitates the correct binding to DNA, while increased condensation of the sperm chromatin is followed by dephosphorylation.

During the sperm passage in the epididymis, cross-linking between cysteine residues of protamine disulfide bonds enhances the stabilization of the nucleoprotamine complex. A healthy mature spermatozoon must decondense this compact structure upon fertilization and reorganize it into a nucleosomal structure. Thus, sperm nuclear DNA organization not only permits the very tightly packaged genetic information to be transferred safely to the egg, but it also allows the transformation of this structure into a more loose configuration so that the genetic information is easily accessible to the developing embryo [37,47,48]. However, it should be mentioned that some regions of the DNA retain a nucleosomal structure throughout spermiogenesis. In humans, about 15% of sperm DNA is associated with histones or other proteins in a typical nucleosomal structure. The rest 85% is in the form of a highly compact nucleoprotamine complex [49-52].

Both transition proteins and protamines are products of unique genes expressed only in male germ cells [53]. The protamines are small, basic proteins that package DNA into a volume twenty fold smaller than the nucleus of somatic cells [54]. Most mammals have only one protamine gene but mice, humans and few other mammals have two sets for each one of protamines P1 and P2. From the human sperm nucleus a group of four protamines (HP1, HP2, HP3, and HP4) have been isolated: protamine HP1, member of the P1 family and protamines HP2, HP3, HP4, members of the P2 family. Protamine P2 performs an essential function in male fertility [55]. Both protamines are expressed in roughly equal quantities [56]. The mean P1/P2 ratio in human sperm is approximately 1.0 [54,57]. Altered P1/P2 ratios have been shown in the sperm from some infertile men as well as a P2 deficiency in mature sperm, whereas protamine abnormalities are rarely seen in the sperm from fertile men [56,58-62].

Overall, this means that, persistence of chromatin nicks in the nuclear DNA of the ejaculated spermatozoa may be a sign of alterations in the repair mechanism during protamination and/or an incomplete maturation process during chromatin condensation [24]. In fact, elevated or diminished protamine 1/protamine 2 ratios have been observed in some infertile men and are often associated with severe spermatogenesis defects [63]. Recently, it has been suggested that evaluation of the P1/P2 ratio may represent a more accurate sperm functional assay among others [64]. Abnormal protamine expression has been associated with low sperm counts, decreased sperm motility and morphology, diminished fertilization ability and increased sperm DNA damage [57,58]. In some cases of human male infertility P2 is completely absent [61] or the normal ratio of the two families of protamines is altered [56,59]. In addition, reduced protamine P2 levels are in some cases associated with high amounts of P2 precursor molecules [61]. Furthermore, disruption of sperm nuclear compaction alters either P2 or P1 gene and the subsequent processing from precursors molecules to mature forms, ensuing in defective sperm function. [65].

### Apoptosis

More than 35 years ago, Kerr *et al *[66], established that apoptosis is a physiological mechanism of controlled cell elimination, complementary but opposite to mitosis, necessary for the development of the definite cell populations and the maintenance of homeostasis of the organism during adulthood. This highly conserved process, also called programmed cell death (PCD), can be initiated or inhibited by a variety of environmental stimuli [66]. Germline cell death is required during normal development for proper formation of gametes and serves to control excessive germ cells number by eliminating the surplus during differentiation. In addition, it can occur in response to checkpoints and be involved in the removal of dangerous or damaged cells [67]. PCD in the germline often displays apoptotic characteristics, such as chromatin condensation, membrane blebbing, release of cytochrome c from mitochondria, formation of apoptosome, an Apaf-1/caspase-9 complex, resulting in further downstream caspase activation, generating substrate cleavage, endonuclease activation and DNA fragmentation [68].

#### Apoptosis in the seminiferous epithelium

Germ cell death occurring during normal spermatogenesis in mammals has been identified for more than a century and estimated to be responsible for the loss of up to 75% of the potential spermatozoal number [69-71]. In fact, one part of A2–A4 spermatogonia is eliminated by apoptosis and only 25% of the theoretically expected number of spermatocytes I is produced from the original population of spermatogonia A1. Moreover, selective death of spermatocytes and spermatids frequently occurs resulting in the elimination of 20% of these cells. Thus, it seems a natural conclusion that programmed cell death must play an important role in spermatogenesis. However, only after the application of *in situ *3'-end labeling techniques to identify DNA fragmentation in each cell type of the seminiferous epithelium was spontaneous germ cell death recognized as being due to apoptosis [72-74]. Several methods such as Hoechst 33258 DNA staining, TUNEL assay, flow cytometric annexin-V binding, immunohistochemical detection of apoptotic markers and others have been employed to identify apoptosis in spermatogonia, spermatocytes and spermatids in the testis of men with normal spermatogenesis, in patients with non-obstructive azoospermia as well as in the ejaculated spermatozoa [13,75,76]. Testicular spermatozoa have been reported to exhibit less DNA damage than those from the ejaculated sample [77].

#### Apoptosis in ejaculated spermatozoa

Both apoptosis and necrosis are observed in ejaculated human sperm [23,26,78]. However, it is uncertain whether ejaculated sperm retains the ability to activate the apoptotic cascade or whether the detected apoptotic markers in spermatozoa are, simply, the expression of an apoptotic process that has began before the event of ejaculation [26]. Moreover, during *in vitro *processing, spermatozoa can not enter in the apoptotic pathway and they are eliminated by necrosis [77]. Gorczyca parallels DNA fragmentation in sperm to the apoptotic cleavage in the linker (internucleosomal) sites seen in somatic cells. But such regions are not present in the ejaculated sperm chromatin and DNA breakage can not attributed to an apoptotic event [79,80].

Apoptotic spermatozoa, observed in the conventional electron microscopy, present the typical morphological characteristics of apoptosis seen in somatic cells [81]. These characteristics, involving nuclear and cytoplasmic elements, are consistently seen in the spermatozoa of fertile men, though in reduced numbers, as well as in these produced by subfertile patients. Nevertheless, a significant correlation between spermatozoal DNA fragmentation and apoptotic features has not been established [79]. Similarly, no association was found between DNA fragmentation and other apoptotic markers such as Fas-receptor, bcl-x, p53 and caspaces, usually seen in somatic cells, although these markers have also been detected in the ejaculate of subfertile men [26,79,82,83].

**Figure 1:**
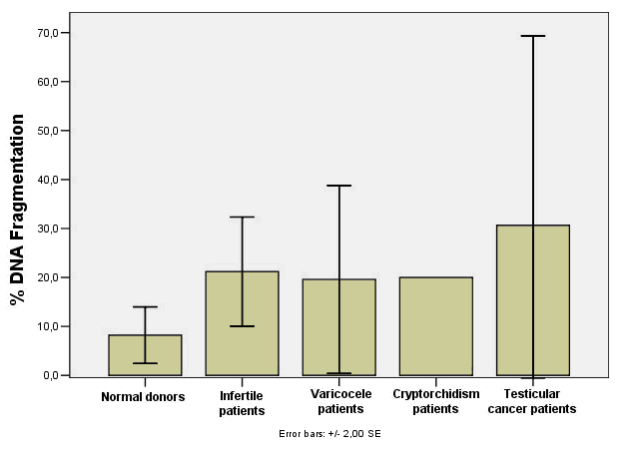
**Frequency of DNA fragmentation in human ejaculated spermatozoa**. The percentage of DNA fragmentation in the above groups was determined using the terminal deoxynucleotidyl transferase mediated dUTP-nick end labeling (TUNEL) assay with or without Hoechst 33258 DNA staining. The arithmetic means of each group were obtained by adding the group's means, when reported by the cited studies, and dividing the sum by the number of the cited studies that evaluated that group. The Standard Error of each group shown in the graph demonstrates the variability between the results of the different studies. All five studies evaluated normal donors [13, 81, 175, 181, 182]. Three of the studies also assessed a group of infertile patients [13, 175, 181], two evaluated varicocele patients [81, 182], one evaluated cryptorchidism patients and two assessed testicular cancer patients [13, 81].

The percentage of spermatozoa with DNA fragmentation in normal donors and different groups of patients is illustrated in figure 1. Gandini et *al*. found a statistically significant reduction (P < 0.001) of the percentage of apoptotic spermatozoa in the semen of healthy men after swim-up compared to the raw sample (1.2% ± 0.7% after swim-up compared to 2.5% ± 1.2% seen in fertile donors) [13]. They observed a statistically significant increase of DNA fragmentation in the semen of patients suffering from various pathologies including oligoasthenoteratozoospermia (OAT), Hodgkin's disease and testicular cancer [13]. Furthermore, it is known that patients suffering from Hodgkin's disease, seminoma or testicular embryonic carcinoma, have spermatozoa exhibiting a high level of DNA fragmentation even before radiation therapy and chemotherapy, which often renders them completely sterile [13,82,84].

### Oxidative stress

The primary source of ROS in seminal plasma is spermatozoa themselves as well as polymorphonuclear leukocytes. Low levels of ROS are required for normal sperm function, capacitation and acrosome reaction [85,86]. In contrast, defective spermatozoa and increased numbers of infiltrating leukocytes, mainly from inflammation at the level of testis, epididymis or the prostate, generate high levels of ROS, exceeding the protective capacity of the seminal fluid [3,87-89]. The ensuing oxidative stress increases the possibility of DNA damage, which can result to sperm dysfunction due to poor sperm quality, loss of capacity to undergo the acrosome reaction, difficulty in the fusion of the sperm with the oocyte and diminished fertility, both *in vitro *and *in vivo *[3,87,88].

The peroxidation of unsaturated fatty acids in the sperm membranes and DNA fragmentation are implicated in the mechanism by which ROS cause DNA damage and disrupt sperm function [90,91]. The analyses of markers for oxidative stress and apoptosis showed a significantly positive correlation between ROS production, oxidative stress and DNA damage (fragmentation and presence of single stranded DNA) [32]. Mitochondria, as the basic source of ROS, are involved in the activation of pro-apoptotic molecules, DNA fragmentation and apoptosis. While oxidative damage of the mitochondrial DNA leads to reduced production of ATP affecting reproductive capacity, nuclear DNA damage is detrimental to the morphology and function of the sperm [92]. Lopes *et al*. have shown that ROS can be the cause of increased DNA fragmentation and poor-quality semen samples with a higher percentage of spermatozoa with fragmented DNA than normal fertile samples, while pre-treatment with antioxidants can reduce this DNA damage [20]. It is not clear why defective human spermatozoa generate high levels of ROS, although an extra-mitochondrial activity, emanating, probably, from the exposure to the unsaturated fatty acids cannot be excluded [93].

#### Genital tract infections and varicocele

Genital tract infections may cause spermatozoal DNA damage and have been associated with increased levels of ROS [3]. Varicocele constitutes a serious cause of male infertility although it is, also, present in 15% of men who procreate [94]. Though the impact of this disease on spermatogenesis is well documented [95] the aetiological mechanisms are not yet very clear. Infertile men with varicocele compared to normal controls which had initiated a natural pregnancy, showed statistically significant higher levels of spermatozoal DNA damage, due to high levels of ROS and elevated intratesticular temperature [96]. More precisely, they showed a higher DFI (calculated by the sperm chromatin structure assay-SCSA) and a significant increase of ROS (evaluated by chemiluminescence), in contrast to the lower total antioxidant capacity (TAC) (assessed by enhanced chemiluminescence) [95]. Besides infertile men, analogous scores of ROS and TAC, have been reported in fertile donors with a clinical diagnosis of varicocele [97,98]. Not only SCSA evaluation, but also an estimation of the apoptotic index (AI) by the TUNEL assay, confirmed the increase of the percentage of spermatozoa with DNA damage in patients affected by varicocele compared to normal fertile men [99]. More recently, the SCD (Sperm Chromatin Dispersion) test has also been used to discriminate different levels of DNA fragmentation in semen from patients with various grades of varicocele compared to fertile donors [100]. ROS may also be generated inside the seminiferous tubules by the cytoplasmic droplets retained in immature spermatozoa [101] and this seems to be a common feature in the sperm samples from infertile men with varicocele [102,103]. Nevertheless, it could be interesting to add that varicocelectomy diminishes the percentage of spermatozoa with damaged DNA (evaluated as DD by flow cytometry of acridine orange-treated spermatozoa), as reported by Zini et *al*. [104]. Finally, the increase of intratesticular temperature is another cause of DNA damage, in patients with varicocele. In fact, it has been shown that testicular hyperthermia, both direct or indirect, can cause DNA damage via an increase in the histone:protamine ratio [12,105].

### Age and spermatozoal DNA damage

Advancing paternal age has been associated with a variety of anomalies, such as diminished semen quality, numerical and structural chromosomal abnormalities, that can decrease reproductive capacity and fertility and increase the frequency of spontaneous abortions [90]. Although these age-related changes in the male reproductive system are universally recognized, the question of declining fecundity with male age remains controversial [90,106,107]. Moreover, several studies have shown that older men seem to produce more spermatozoa with damaged DNA [82,91,108,109], emanating from the three main potential sources: oxidative stress, abortive Fas-mediated apoptosis or abnormal chromatin packaging [110].

#### Does DNA damage appear more frequently in the sperm of older men?

Recently, Singh *et al*. found an increase in sperm double-stranded DNA breaks and a decrease in apoptosis with age by using neutral microgel electrophoresis (Comet) and DNA diffusion assay [91]. The percentage of sperm with highly damaged DNA, comet extent, DNA break number and other comet measures were significantly higher in men aged 36–57 years compared to those aged 20–35 years. However, the percentage of apoptosis was significantly lower in the older group [91]. Furthermore, in a histomorphometric study of 36 men, aged from 61 to 102 years and 10 young men from 29 to 40 years old, evaluation of the aneuploidy rate and DNA fragmentation revealed a decrease in the number of germinal and Sertoli cells with age, although, besides individual variations, spermatogenesis was possible until a very advanced age (95 years). When spermiogenesis was arrested, an increased aneuploidy rate in postmeiotic cells was observed, whereas apoptosis in the spermatozoa was not increased with age [111]. In addition, several studies have demonstrated an age-related increase in sperm cells with double-stranded DNA breaks or poor chromatin packaging [91,109,112-114]. This age-related effect may happen as older men produce more sperm with fragmented DNA due to a higher exposure to oxidative stress in their reproductive tracts [32,115]. Oxidative stress can damage sperm DNA and mitochondrial and nuclear membranes [116]. Furthermore, the apoptotic function during spermatogenesis may be less effective in older males resulting in the accumulation of more spermatozoa with fragmented DNA [117,118]. These observations on the differential effects of age on genomic damage are consistent with the recent findings of Wyrobek *et al*. [109], who reported age-related effects on DNA fragmentation and achondroplasia mutations but not aneuploidy, Apert syndrome mutations or sex chromosome ratio.

**Figure 2:**
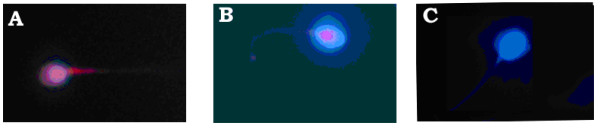
**TUNEL assay**. TUNEL-positive nuclei (with double-strand nuclear DNA fragmentation) of spermatozoa as represented by the intense (A) and dull (B) Texas red fluorescence in the nuclear region. The healthy nuclei (without DNA fragmentation) are stained blue with DAPI (C) used as counterstain.

**Figure 3:**
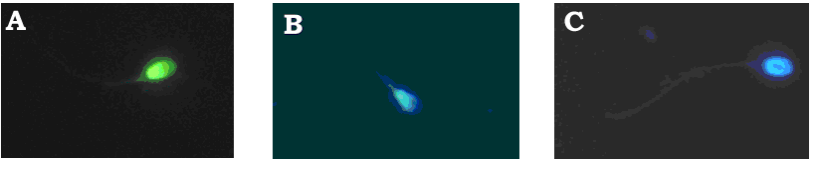
**CMA_3 _staining**. Two types of staining patterns were identified, bright and dull yellow fluorescence of the sperm nuclei (abnormal chromatin packaging) (A, B) and blue staining with DAPI in the healthy nucleus (normal chromatin packaging) seen in C.

**Figure 4:**
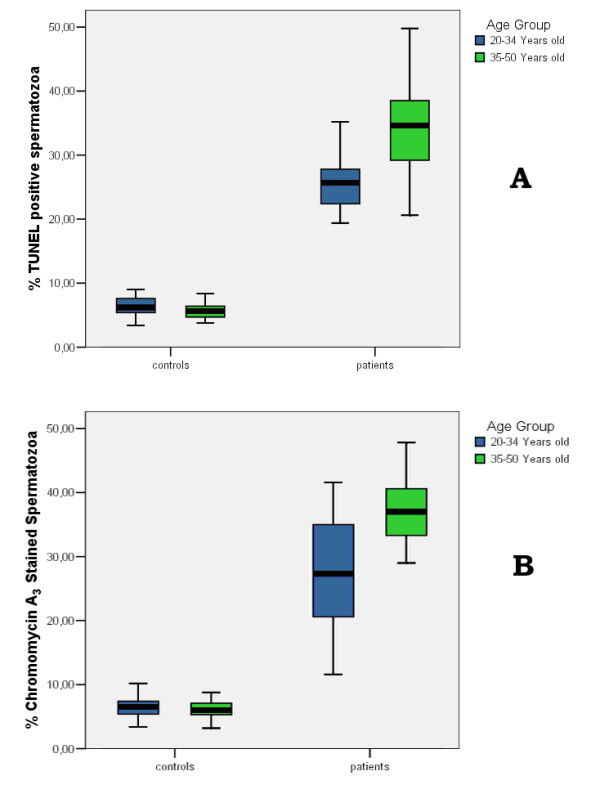
**The effect of age on DNA fragmentation and chromatin packaging. **DNA fragmentation was assessed using the TUNEL assay, while chromatin packaging was evaluated using the chromomycin A_3 _(CMA_3_) staining. Sixty one oligoasthenoteratozoospermic patients were divided into two age subgroups (20–34 years old, n = 30; 35–50 years old, n = 31). Forty nine healthy fertile controls were also divided according to their age (20–34 years old, n = 26; 35–50 years old, n = 23). In the control group, the differences observed between the two age subgroups were not statistically significant (P > 0.05; Figure 4A, 4B). On the other hand, the older patient subgroup demonstrated a significantly higher percentage of TUNEL positive (P < 0.001; Figure 4A) and CMA_3 _stained (P < 0.001; Figure 4B) spermatozoa compared to the younger patient subgroup.

In our laboratory, we used the TUNEL assay (Figure 2) and Chromomycin A3 staining (Figure 3) to assess the aging effects on the percentages of DNA fragmentation and chromatin packaging respectively in oligoasthenozoospermic patients (Figures 4). Sixty one oligoasthenoteratozoospermic patients were divided into two age subgroups (20–34 years old, n = 30; 35–50 years old, n = 31). Forty nine healthy fertile controls were also divided according to their age (20–34 years old, n = 26; 35–50 years old, n = 23). In the control group, the differences observed between the two age subgroups were not statistically significant (P > 0.05; Figure 4A, B). On the other hand, the older patient subgroup demonstrated a significantly higher percentage of TUNEL positive (P < 0.001; Figures 2, 4A) and CMA3 stained (P < 0.001; Figures 3, 4B) spermatozoa compared to the younger patient subgroup. In addition, the older donors (35–50 years old, n = 23) showed significantly lower percentages of TUNEL positive and CMA3 stained spermatozoa compared to the younger patient subgroup (20–34 years old, n = 30; P < 0.001). The younger patient subgroup (20–34 years old, n = 30) demonstrated significantly higher percentages of TUNEL positive and CMA3 stained spermatozoa compared to the younger donor subgroup (20–34 years old, n = 26; P < 0.001).

Indeed, the reproductive potential of older couples is compromised. The above results attest that, in addition to the increased abnormalities in conventional semen parameters [115] and the reduced DNA repair capabilities, other factors are also operating in aged males. This can also be attributed to an accumulation of DNA-damaged spermatozoa due to a deficient apoptotic machinery. Thus, the decreased elimination and subsequent accumulation of defective spermatozoa, concomitantly with poor DNA integrity and abnormal chromatin packaging were confirmed to be operational in the older male. Many of these couples seek specialized help from *in vitro *fertilization (IVF) clinics. Following this procedure, the natural selection of "healthy" sperm will be bypassed and the possible use of defective spermatozoa may negatively affect the ART outcome. In some ART centers, semen samples are prepared immediately prior to their use, in order to overcome the negative effect of a prolonged time of incubation of a density gradient selected sperm.

### Influence of sperm DNA damage on reproductive outcome

Male infertility is associated with poor sperm DNA integrity and it has been suggested that these DNA abnormalities may affect procreation in couples having natural intercourse and in those treated by IUI, IVF and intracytoplasmic sperm injection (ICSI) [119-122]. In addition, fragmented DNA is a more common finding in couples having a history of recurrent miscarriages than in those attempting pregnancy via IVF or donor insemination [123]. There is also strong clinical evidence that the combination of increased sperm DNA damage with abnormalities in standard semen parameters can have an obvious impact on the reproductive potential [121,122,124,125]. Furthermore, for a threshold value of DNA fragmentation above 10%, a significant negative correlation to the fertilization rate has been reported by Benchaib *et al*. [126].

However, several studies have examined the possible influence of sperm DNA damage on the reproductive outcome of both standard IVF and IVF/ICSI, showing no consistent relationship [12,119-122,124,126-134]. Since the embryonic genome is not expressed until after the second cleavage division, it is logical to assume that sperm DNA damage does not affect fertilization or embryo development [12,135]. Furthermore, no consistent relationship seems to exist between sperm DNA damage and embryo quality after ICSI (approximately half of the studies have shown an adverse effect) [12]. High levels of sperm DNA damage, however, are inversely correlated to pregnancy rates in most of the studies [12,119-122,124,126-134].

The influence of DNA fragmentation index (DFI) on the fertilization rate and pregnancy outcome varies from study to study. In one investigation, the highest DFI found among fertile donors was 24% and has been used for the classification of infertile men into high (> 24%) and low (≤ 24%) DNA damage groups [2]. In cases involving ICSI, the fertilization rate decreased when the DFI was elevated but, nevertheless, term pregnancies have been obtained even with high sperm DFI (> 30%) [8]. Furthermore, Virro *et al*. found that men with high levels of DNA fragmentation (≥ 30% DFI) had a significantly lower chance of initiating a chemical pregnancy compared to men with < 30% DFI, whereas no relationship between high DNA stainability (HDS) and chemical pregnancies, spontaneous abortions or ongoing pregnancies was observed [121]. Boe-Hansen *et al*. found that by using spermatozoa with a DFI > 27%, the probability of a successful pregnancy may be reduced, although full-term pregnancy can still be achieved [136]. Evenson and Wixon reported that semen samples containing ≥ 30% spermatozoa with fragmented DNA were found inappropriate to achieve pregnancy [16]. In another study, the prognostic value of sperm DNA fragmentation levels in predicting IVF and ICSI outcome was determined in 85 couples. An inverse correlation between the percentage of spermatozoa with fragmented DNA and fertilization rate, synchronization of the "nucleolar precursor bodies" pattern in pronuclei, blastocyst development and embryo quality was reported [137].

Overall, DFI levels higher than 15% can multiply the risk of miscarriage fourfold (37.5% versus 8.8%) and worsen the prognosis for pregnancy [138]. Moreover, in the largest study on the predictive value of SCSA in relation to the outcome of ART by Bungum *et al*. it was shown that if DFI exceeds 30%, ICSI should be the preferred approach even in cases where traditional sperm parameters are normal [139]. In this same study it was also reported that in almost 20% of the patients DFI was found > 30%, although semen parameters fulfilled the criteria for either IUI or IVF. Furthermore, no statistically significant association between high DFI and early pregnancy loss was observed, contrasting previous reports that reported an increased risk of embryonic loss in pregnancies achieved by the use of semen samples with high rates of DNA breaks [121,123], However, the possibility that DFI levels higher than 60% might be associated with an increased risk of early pregnancy loss could not be excluded.

## Methods used to assess DNA damage

### Detection of DNA fragmentation

Many assays, both direct and indirect, are used for detecting DNA breaks (Table 1). Direct methods include: i) the deoxynucleotidyl transferase-mediated dUTP nick end labeling (TUNEL) assay [22,23,84], ii) the single-cell gel electrophoresis assay (Comet assay) [140-142] and iii) the *in situ *nick translation assay with or without sperm decondensation [19,143], whereas the indirect methods include: i) Acridine Orange Technique (AOT), first introduced by Teyada RI *et al*. [144] and ii) Sperm Chromatin Structure Assay (SCSA) [145].

**Table 1:** Sperm chromatin damage assays

**Assays for the evaluation of sperm chromatin maturity/DNA integrity**	**Assay Principle**	**References**
Single-cell gel electrophoresis (Comet Assay)	Single and double-strand breaks, evaluates DNA integrity	[140-142, 152-154]
Terminal deoxynucleotidyl transferase-mediated dUTP- nick end labelling – TUNEL	DNA fragmentation, single and double-strand breaks	[22, 23, 80, 175, 183]
Acridine Orange Technique (AOT)	Distinguish between single and double stranded DNA	[144, 147, 148, 170, 184, 185]
Sperm Chromatin Structure Assay (SCSA)	Acid DNA denaturation	[145, 151]
*In situ *nick translation	Single-strand DNA breaks	[19, 143, 150]
Sperm chromatin dispersion test (SCD)	Determines the susceptibility of sperm DNA to acid denaturation	[163]
DNA diffusion assay	Alkali-labile sites in alkaline conditions yield low- molecular-weight DNA fragments	[164]
Acidic Aniline Blue	Stains lysine residues of persisting histones	[60, 166, 167, 169, 170]
Toluidine blue stain	DNA structure and chromatin packaging, incorporates in the damaged dense chromatin	[165, 185]
Chromomycin A_3 _– CMA_3_	Indirect visualization of nicked denatured DNA	[149, 171, 172]

### Principle and use of each technique

The TUNEL assay detects both single- and double-stranded DNA fragments by labelling the 3'-OH termini with a fluorescently labelled nucleotide (dUTP) in an enzymatic reaction driven by terminal deoxynucleotidyl transferase (TdT) [79]. This technique was first described by Gavrieli *et al*. [146] and used to detect DNA strand breaks in mammalian spermatozoa. The percentage of spermatozoa with fragmented DNA is determined by direct observation using an epifluorescence microscope or by using flow cytometry. Hence, the TUNEL technique has been used in numerous studies [9,13,32,68,147,148]. In one of them it has been demonstrated that the fractions of low sperm motility from ejaculates of infertile men showed a relatively high proportion of cells with DNA fragmentation [31]. Another study, undertaken to analyze the possible relationship between ART failure and sperm DNA fragmentation, revealed high percentages of spermatozoa with fragmented DNA often found in cases of repeated ART failures [148]. Finaly, Sergerie *et al *demonstrated that infertile patients have a higher mean level of DNA fragmentation compared to men of proven fertility [9].

The nick translation technique measures the incorporation of radiolabelled thymidine in the 3' ends of broken DNA strands by monitoring the activity of the DNA polymerase I enzyme and revealing by radioautography the "laddering" pattern of the resulting fragments. Alternatively, nucleotides labeled with biotin or digoxigenin can also be used for incubation with DNA polymerase I. The template-dependent enzyme (DNA polymerase I) in the presence of DNA nicks, by virtue of its 5'-3' exonucleotic activity, catalyzes the movement of the nicks along the double helix [149,150].

The Comet assay involves embedding of spermatozoa in agarose on a glass slide, applying electrophoresis and evaluating DNA migration in comet tails with a software program [145,151]. It is a simple, rapid and sensitive technique for the assessment and quantification of DNA damage in individual cells, first introduced by Östling and Johanson in 1984 [152] as a neutral assay in which the lysis and electrophoresis were done under neutral conditions. Subsequently, Singh *et al*, introduced a microgel technique involving electrophoresis under alkaline (pH 13) conditions for detecting DNA damage in single cells [153] and Haines *et al*. detected DNA damaged spermatozoa after *in vitro *irradiation [154].

Regarding the indirect methods, both the acridine orange technique (AOT) and the sperm chromatin structure assay (SCSA) are used to measure the susceptibility of sperm DNA to acid-induced denaturation [144]. Normal double-stranded DNA stains green and single-stranded DNA stains red in AOT [155,156], a simple microscopic procedure that comes with its own set of problems, while SCSA uses flow cytometry [145,151]. The percentage of DNA fragmentation, also referred to as DNA Fragmentation Index (DFI) is yielded by the ratio of red/red+green. The percentage of sperm showing > 56% red fluorescence is considered as the "cut-off" value to characterize an abnormal chromatin status [157-159]. *In situ *detection of sperm DNA damage using automated instrumentation (flow cytometers) was described for the first time in 1980 [160]. Evenson *et al*. [145] later refined the SCSA protocol. Although AOT and SCSA are based on the same principle and use the same chromatin intercalating metachromatic dye acridine orange, several differences between them have been reported [161]. Furthermore, SCSA and AOT have been compared by running the 2 techniques on the same human samples and in both cases, the results were not significantly associated [162].

Recently, novel clinical tests for DNA fragmentation and apoptosis were introduced, such as the Sperm Chromatin Dispersion test (SCD) that determines the susceptibility of sperm DNA to acid denaturation and the DNA diffusion assay [163,164]. The SCD test principle is that sperm with fragmented DNA fails to produce the characteristic halo of dispersed DNA loops observed in sperm with double-stranded DNA following acid denaturation and removal of nuclear proteins. The DNA diffusion assay is a simple and reproducible test based on the principle that apoptotic cells have numerous alkali-labile sites which, once exposed to alkaline conditions, yield low-molecular-weight DNA fragments. These pieces are easily diffusable in the agarose matrix, giving a characteristic pattern of DNA gradient, with the appearance of a hazy halo around the apoptotic cells.

Several researchers have shown that sperm DNA denaturation, as measured by the SCSA test, is strongly correlated with other DNA damage biomarkers such as TUNEL and Comet assays [10,21,140]. Nevertheless, the SCSA analysis is considered by some investigators to be the most reliable test, needed in a clinical setting to help manage a couple's infertility treatment, while the Comet and TUNEL assays lack the precision, consistency and reproducibility necessary to this end [16]. Conversely, others believe that DNA fragmentation can be successfully detected with SCSA, TUNEL, and SCD yielding similar results, in respect to accuracy and sensitivity [162].

### Sperm nuclear maturity tests

These tests measure the sperm chromatin packaging quality and the protamine content directly, through protamine extraction and polyacrylamide gel electrophoresis (PAGE), or indirectly. Toluidine blue staining was first described by Krzanowska H. in 1982 [165]. Assays using aniline blue or toluidine blue are applied to detect the lysine-rich nucleoproteins (histones) and, therefore, give an indication of the presence of lower amounts of protamines in the sperm nucleus [60,166]. Asthenozoospermic semen samples show increased percentage of aniline blue cells compared to normozoospermic samples [167]. Acidic aniline blue has also been correlated with differences in sperm nuclear morphology in sperm donors and in infertile patients [168,169]. Furthermore, the persistence of lysine-rich nucleoproteins revealed by acidic aniline blue staining correlates positively with acridine orange [170] and chromomycin A_3 _staining [171].

A polymerase inhibitor, CMA_3_, is used in another indirect approach for the evaluation of normal protaminosis. This test was fist described by Hayasaka T and Inoue Y in 1969 [172]. Based on the *in situ *competition between protamine and CMA_3_, this assay is inversely correlated with the protamination state of spermatozoa [173]. This means that incorporation of CMA_3 _can prevent the accessibility of DNA polymerase I to the DNA and, almost none of the CMA_3_- negative spermatozoa present nicked DNA, as CMA_3 _is unable to access DNA in the presence of protamines and normally formed disulphide bonds [24,149,174]. CMA_3 _staining is evaluated by distinguishing between spermatozoa nuclei with bright or dull yellow staining (CMA_3 _positive) compared to spermatozoa nuclei counterstained blue with DAPI (CMA_3 _negative) as shown in figure 4. Normozoospermic men demonstrate a lower percentage of spermatozoa with DNA damage and CMA_3 _positivity compared with oligozoospermic and teratozoospermic men. Moreover, patients that do not establish a pregnancy show an increased percentage of DNA-damaged spermatozoa [129,174].

The hypothesis that the presence of DNA damage in mature spermatozoa is correlated to poor chromatin packaging [19] is supported by the results of Mantas *et al*. [175], who showed a significant positive correlation between DNA fragmentation (TUNEL assay) and chromatin packaging (CMA_3 _staining) in men with low semen quality. Correlations between CMA_3 _staining, sperm morphology, fertilization and assisted reproduction outcome have been found in patients undergoing routine IVF, subzonal insemination (SUZI) or ICSI [176-179]. Thus, CMA_3 _is a useful tool for evaluating infertile patients as it will stain decondensed, protamine-depleted spermatozoa. However, CMA_3 _staining cannot discriminate whether the potential protamine deficiency is due to a lack of P1, P2 or a combination of both [62]. This disadvantage can be resolved by measuring protamines P1 and P2 directly by gel electrophoresis. In fact a significant negative correlation of the fertilization rate with the protamine deficiency and the P1/P2 ratio was found using this approach [180].

## Conclusion

Analysing the impact of specific biomarkers of protaminosis and sperm DNA integrity, it becomes apparent that their use as indicators associated with normal chromatin packaging and normal semen parameters can assist in eliminating the risk of using spermatozoa with defective DNA and, thus, lead to the improvement of male fertility, successful conception and pregnancy outcome. Although, an important level of sperm DNA damage can also be detected in fertile men this is more pronounced in infertile men attending reproductive clinics, especially among older men whose reproductive potential is compromised. Beyond the increased abnormalities in conventional semen parameters and the reduced DNA repair capabilities, this can also be attributed to an accumulation of DNA-damaged spermatozoa due to deficient apoptotic machinery. Thus, the decreased elimination and subsequent accumulation of defective spermatozoa, concomitantly with poor DNA integrity and abnormal chromatin packaging, result in a sperm of poor quality. Injecting these abnormal spermatozoa into oocytes will probably result in failure of sperm decondensation and fertilization, as there appears to be a threshold value of sperm DNA damage beyond which embryo development and subsequent pregnancy outcome are impaired. Therefore, detection of damaged DNA in spermatozoa needs to be conducted along with standard semen analysis. Assessment of the results from various techniques on this issue could be a very efficient diagnostic tool for the evaluation of the underlying pathologies. In addition, large-scale studies in different clinical settings may be useful to explain the presence of defective DNA in patients with unexplained infertility and to determine the effects of sperm DNA damage on the outcome of ART.

## Abbreviations

AOT - Acridine Orange Technique.

ART - Assisted Reproduction Technology.

CMA_3 -_ Chromomycin A_3_.

DD - DNA Denaturation.

DF - DNA Fragmentation.

DFI - DNA Fragmentation Index.

dUTP - Labeled Nucleotide.

FISH - Fluorescence *in situ *Hybridization.

FITC - Fluorescein Isothiocyanate.

HDS - High DNA Stainability.

ICSI - Intracytoplasmic Sperm Injection.

IVF - *in vitro *fertilization.

OAT - Oligoasthenoteratozoospermia.

PCD - Programmed Cell Death.

QPCR - Quantitative Polymerase Chain Reaction.

ROS - Reactive Oxygen Species.

SCD - Sperm Chromatin Dispersion.

SCSA - Sperm Chromatin Structure Assay.

SSB - Single-strand breaks.

SUZI - Subzonal Insemination.

TAC -  Total Antioxidant Capacity.

TdT - Terminal Deoxynucleotidyl Transferase.

TNPs - Transition Proteins.

TUNEL - Terminal deoxynucleotidyl transferase mediated dUTP-nick end labeling.

WHO - World Health Organization.

## Competing interests

To the authors' best knowledge, no competing interests of any nature arise from the current publication

## Authors' contributions

The three authors have drafted the article, revised it critically and gave final approval of the version to be published. In addition, all the images of the manuscript were prepared using image and statistics analysis software by PM. All authors read and approved the final manuscript.
